# Defined microbiota transplant restores Th17/RORγt^+^ regulatory T cell balance in mice colonized with inflammatory bowel disease microbiotas

**DOI:** 10.1073/pnas.1922189117

**Published:** 2020-08-18

**Authors:** Graham J. Britton, Eduardo J. Contijoch, Matthew P. Spindler, Varun Aggarwala, Belgin Dogan, Gerold Bongers, Lani San Mateo, Andrew Baltus, Anuk Das, Dirk Gevers, Thomas J. Borody, Nadeem O. Kaakoush, Michael A. Kamm, Hazel Mitchell, Sudarshan Paramsothy, Jose C. Clemente, Jean-Frederic Colombel, Kenneth W. Simpson, Marla C. Dubinsky, Ari Grinspan, Jeremiah J. Faith

**Affiliations:** ^a^The Precision Immunology Institute, Icahn School of Medicine at Mount Sinai, New York, NY 10029;; ^b^Icahn Institute for Data Science and Genomic Technology, Icahn School of Medicine at Mount Sinai, New York, NY 10029;; ^c^Department of Clinical Sciences, College of Veterinary Medicine, Cornell University, Ithaca, NY 14853;; ^d^Department of Oncological Sciences, Icahn School of Medicine at Mount Sinai, New York, NY 10029;; ^e^Janssen Research & Development, Spring House, PA 19477;; ^f^Janssen Human Microbiome Institute, Janssen Research and Development, Cambridge, MA 02142;; ^g^Centre for Digestive Diseases, Sydney, NSW 2046, Australia;; ^h^School of Medical Sciences, University of New South Wales, Sydney, NSW 2052, Australia;; ^i^Department of Gastroenterology, St Vincent’s Hospital, Melbourne, VIC 3065, Australia;; ^j^Department of Medicine, St Vincent’s Hospital, Melbourne, VIC 3065, Australia;; ^k^Department of Gastroenterology, University of Melbourne, Melbourne, VIC 3010, Australia;; ^l^Concord Clinical School, University of Sydney, Sydney, NSW 2050, Australia;; ^m^Department of Gastroenterology & Hepatology, Macquarie University Hospital, Sydney, NSW 2109, Australia;; ^n^The Dr. Henry D. Janowitz Division of Gastroenterology, Icahn School of Medicine at Mount Sinai, New York, NY 10029

**Keywords:** mucosal immunology, microbiome, fecal microbiota transplant, Th17 cells, regulatory T cells

## Abstract

The building evidence for the contribution of microbiota to human disease has spurred an effort to develop therapies that target the gut microbiota. This is particularly evident in inflammatory bowel diseases (IBDs), where clinical trials of fecal microbiota transplantation have shown some efficacy. To aid the development of novel microbiota-targeted therapies and to better understand the biology underpinning such treatments, we have used gnotobiotic mice to model microbiota manipulations in the context of microbiotas from humans with inflammatory bowel disease. Mice colonized with IBD donor-derived microbiotas exhibit a stereotypical set of phenotypes, characterized by abundant mucosal Th17 cells, a deficit in the tolerogenic RORγt^+^ regulatory T (Treg) cell subset, and susceptibility to disease in colitis models. Transplanting healthy donor-derived microbiotas into mice colonized with human IBD microbiotas led to induction of RORγt^+^ Treg cells, which was associated with an increase in the density of the microbiotas following transplant. Microbiota transplant reduced gut Th17 cells in mice colonized with a microbiota from a donor with Crohn’s disease. By culturing strains from this microbiota and screening them in vivo, we identified a specific strain that potently induces Th17 cells. Microbiota transplants reduced the relative abundance of this strain in the gut microbiota, which was correlated with a reduction in Th17 cells and protection from colitis.

## Significance

Composition of gut microbiota is altered in many human diseases, including inflammatory bowel disease. Some hope that restoring microbiota to a healthy state could help treat such diseases. We have used mice colonized with microbiotas from humans with inflammatory bowel disease to study what happens when these mice receive a microbiota transplant from a set of healthy humans. We find that the mouse gut immune system is changed by microbiota transplants, becoming broadly less inflammatory and protecting mice from colitis. By culturing bacteria from these microbiotas, we identify one strain that induces inflammatory responses in mice and show that it is modified by microbiota transplant. We also show that increases in the density of microbiota following transplant may be antiinflammatory.

The composition of the human gut microbiota is altered in many chronic human diseases. This fact has driven an effort to understand how microbiota contribute to disease and if it represents a target for novel therapies. In the majority of conditions, no consistent association between the disease and any specific commensal species or strain has been made, and the distinction between the microbiota in the healthy and diseased states reflects broader differences in the community structure, diversity, or density (1, 2). In the rare cases where specific species or strains are hypothesized to contribute to disease—for example, adhesive-invasive *Escherichia coli* (AIEC) in Crohn’s disease (3)—targeted therapy using approaches such as phages may be possible (4). In other situations, rational therapeutic approaches are elusive, and the foremost strategy to modulate the microbiota has been fecal microbiota transplantation (FMT).

FMT is effective for treating recurrent *Clostridioides difficile* infection (rCDI) (5), but is also under investigation for the treatment of ulcerative colitis (UC), Crohn’s disease (CD), liver diseases, obesity and metabolic disease, food allergy, graft-versus-host disease, checkpoint colitis, and to improve the efficacy of cancer immunotherapy (6–8). Clinical trials of FMT in UC have suggested some potential for using microbiota-targeted therapy in this setting (9–12), but evidence for efficacy in other indications is currently extremely limited, and there is currently little or no scientific basis for the adoption of FMT as a mainstream therapeutic option outside of rCDI.

As seen in rCDI and some individuals with UC, FMT can restore health to the gut, but the mechanisms remain opaque. Studies in gnotobiotic mice have revealed defined gut microbiota manipulations that modulate the proportion or function of specific cells, and these data are aiding the development of candidate live biotherapeutic products designed to specifically target certain pathways or cells, for example, increasing the proportion of regulatory T cells (13, 14). However, to develop microbiota-targeted therapeutics for broad use, it is imperative to understand the specifics of how microbiota manipulations alter host processes and phenotypes in predictable ways. Only by doing this can we hope to achieve the maximum potential benefit of these approaches while exposing treatment recipients to the minimum of risk, as we demand of all other drugs and therapeutics in common use.

This is an extremely complex problem as numerous aspects of the immune system are shaped by the gut microbiota. This is evident in gnotobiotic mice, where the gut immune system is reproducibly altered by microbiotas of different composition (15, 16). However, even under tightly controlled conditions in gnotobiotic mice, predictably linking microbiota composition to host phenotype is challenging. Certain components of the intestinal microbiota have defined immunomodulatory properties in the lamina propria when transferred to mice. For example, segmented filamentous bacteria (SFB) and some strains of *E. coli* induce T helper (Th)17 cells (17, 18), certain strains of *Klebsiella* can induce Th1 cells (19), some strains of *Bacteriodes ovatus* induce high fecal IgA (20), and specific clostridial strains can induce regulatory T (Treg) cells (14, 16, 21). The context within which individual microbes colonize a host can alter their function. For example, immunomodulatory properties of individual strains can be altered in an inflamed host or when cocolonized with different organisms (20, 22). Thus, the immune landscape of the gut is influenced by the presence of specific strains and the compositional context of the broader microbiota, making the rational design of therapies targeting the microbiota challenging.

In ex-germ–free mice colonized with different complex human microbiotas from numerous human donors, population-scale associations can be made between characteristics of the microbiota donors and the phenotype transferred to mice. For example, we have previously observed that microbiotas from donors with inflammatory bowel disease induce more gut Th17 cells and fewer RORγt^+^ Treg cells than microbiotas from healthy donors (HDs) (15). These altered immune populations in wild-type mice are highly predictive of disease severity in a model of colitis using ex-germ–free mice colonized with the same IBD and non-IBD gut microbiotas, implying a strong functional relevance of these cells. These results suggest that different compositions of gut microbiota set stable immune landscapes in the gut lamina propria and alter disease susceptibility. Specifically, the inflammatory tissue immune program in ex-germ–free mice colonized by the gut microbiota of individuals with IBD and characterized by high Th17 cells and low RORγt^+^ Treg cells therefore represents a colitis-susceptible state that is potentially correctable by gut microbiota manipulation.

We established a gnotobiotic mouse experimental system to evaluate potential live bacterial therapy for IBD. This system allowed us to track recipient and donor strains during the procedure and monitor immunological changes in the host following treatment. Germ-free mice were initially colonized with a defined, cultured microbiota obtained from one of three donors with IBD, followed 3 wk later by a transplant with one of five defined consortia of HD-derived microbes. In testing 14 different IBD–HD combinations, we find that the inflammatory gut immune landscape induced by human IBD microbiotas is reversible and that transplantation with HD microbiotas can restore a tolerant immunological state. HD microbiota transplant consistently and reproducibly increased the proportion of gut RORγt^+^ Treg cells in mice initially colonized with IBD donor microbiotas. In mice colonized with an IBD donor that potently induces Th17 cells, transplants reduced the proportion of mucosal Th17 cells. We identify two distinct mechanisms associated with the recovery of gut immune health following transplant. Transplant-induced RORγt^+^ Treg cell induction was associated with increased fecal microbiota density, a measure previously associated with health (1, 23, 24). Where we observed reduced mucosal Th17 cells, this was associated with decreased colonization by a specific strain from the IBD microbiota. Further characterization revealed this strain was required for Th17 induction by this IBD microbiota. We demonstrate that the proinflammatory immune tone driven by IBD microbiotas is reversible by defined microbiota transplant.

## Defined Microbiota Transplant Enables Donor Strain Engraftment

**Fig. 1.**
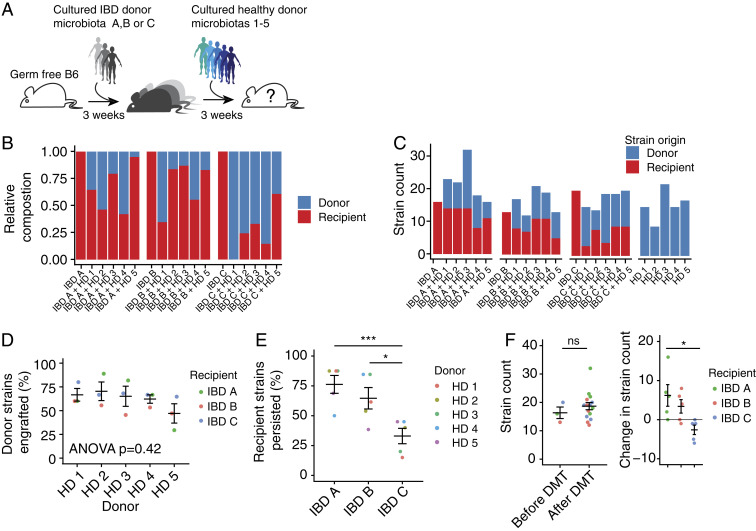
Defined microbiota transplant enables donor strain engraftment. (*A*) Germ-free mice were first colonized with one of three defined IBD donor microbiotas before microbiota transplant with one of five defined HD microbiotas 3 wk later. (*B*) The average contribution of IBD donor- and HD-derived strains to the microbiota 3 wk after transplant as a proportion of the total community. (*C*) The average number of IBD donor- and HD-derived strains that comprise the microbiota 3 wk after transplant. (*D*) The average proportion of strains from each HD that engraft when given as a microbiota transplant to each IBD microbiota-colonized recipient. (*E*) The average proportion of strains from each IBD donor that persist following each microbiota transplant. (*F*) The average total number of strains comprising the microbiota of mice colonized with IBD donor-derived microbiota before and after transplant and the change in the total number of strains following transplant. Plots in *D*–*F* show the mean of each group of mice ± SE. *P < 0.05, ***P < 0.001, ns: not significant by ANOVA with Tukey correction.

To establish a recipient-specific baseline immune tone, we first colonized germ-free C57BL/6 mice with defined, cultured fecal microbiotas (15) isolated from one of three different individuals with IBD (Fig. 1*A* and Dataset S1). Three weeks later, we introduced one of five defined cultured HD fecal microbiota via a single gavage (i.e., a defined microbiota transplant [DMT]) whereupon mice were individually housed to prevent mouse-to-mouse transmission of the DMT. We tested combinations of three IBD and five HDs in a minimum of six mice (14 total combinations) (Fig. 1*A*, Dataset S1, and *Methods*). We focused these experiments on cultured consortia where each species has a complete genome sequence, as this facilitates strain-level tracking of engraftment and downstream identification of immunomodulatory strains.

We performed metagenomic sequencing of DNA isolated from feces of mice before DMT, 3 wk after DMT, and from mice colonized with only the healthy microbiotas used in the DMTs. The HD microbiotas were composed of between 9 and 22 strains (mean 15.6). An average of 59.3% (range: 31.2 to 100%) of the HD strains were detected in the mice 3 wk after DMT (Fig. 1 *B* and *C*). The proportion of strains that were engrafted from each HD was not significantly different (*P* = 0.42, ANOVA; Fig. 1*D*), and there was no difference in the mean proportion of strains that were engrafted from the donors in mice colonized with each IBD donor microbiota (*P* = 0.18, ANOVA; Fig. 1*C*). The three IBD microbiotas comprised 17, 13, and 20 strains, respectively. An average of 58.0% (range: 15.0 to 87.5%) of the strains from the IBD donors remained detectable in the mice following DMT (Fig. 1*E*). No one donor induced a greater loss of IBD microbiota strains than any other (*P* = 0.98, ANOVA), but the proportion of strains from donor IBD C that persisted following transplant was lower than microbiotas from donors IBD A and IBD B, although it is unclear if this was due to compositional differences between the IBD microbiotas or due to variation in the number of strains in each consortium (Fig. 1*E*). On average, the number of strains lost from the microbiotas was balanced by the engraftment of HD-derived strains, and the total number of strains comprising the posttransplant microbiotas was not significantly different from the number of strains detected in the IBD microbiotas before DMT (Fig. 1*F*).

## Modulation of Intestinal Th17 Cells by DMT

**Fig. 2.**
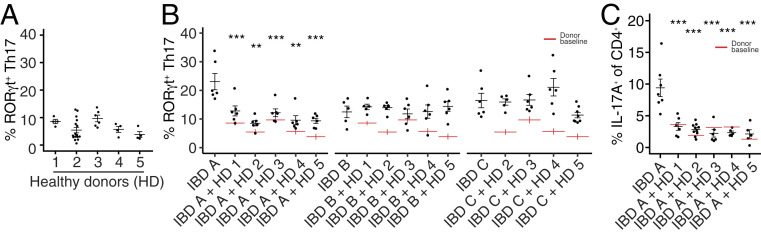
Modulation of mucosal Th17 cells by defined microbiota transplant. (*A*) The proportion of RORγt^+^ Th17 cells (of live CD4^+^FoxP3^−^ cells) in the colon lamina propria of gnotobiotic mice colonized with the five HD-derived microbiotas used as DMT donors. (*B*) The proportion of colon lamina propria RORγt^+^ Th17 cells (of live CD4^+^FoxP3^−^ cells) in groups of mice colonized with each IBD donor alone or 3 wk following DMT with one of the five HD microbiotas. Red lines indicate the proportion of Th17 cells induced by each HD alone, as shown in *A*. (*C*) The proportion of IL-17A^+^ CD4^+^ T cells (of live CD4^+^ cells) in the colon lamina propria of mice colonized with IBD A alone or 3 wk following DMT with one of the five HD microbiotas. Each point represents data from one mouse, and black lines indicate the mean and SE of each group. Red lines indicate the mean proportion of the specified cells induced by each HD alone. **P < 0.01 and ***P < 0.001 as assessed by ANOVA with Tukey correction.

Three weeks after DMT, we assessed the impact of the transplanted microbes on the host intestine tissue resident immune populations in comparison to the impact on mice receiving no transplant that were colonized with either recipient IBD microbiota or HD microbiota alone. We used flow cytometry to profile CD4 T cell populations in the colon and ileum for each donor, recipient, and donor+recipient combination. A hallmark of mice colonized with IBD donor microbiotas is an increase in the proportion of gut Th17 cells, relative to mice colonized with HD microbiotas (15, 18, 25). As demonstrated in multiple animal models, increased Th17 cells are associated with susceptibility to inflammatory disease (26). Therefore, we hypothesize that a rational aim of microbiota-targeted therapy for IBD is to develop interventions that reduce these potentially pathogenic microbiota-induced Th17 cells. With this in mind, we selected as DMT donors HD-derived microbiotas that consistently induced a low proportion of LP Th17 cells (Fig. 2*A*). We measured the proportion of gut Th17 cells in mice colonized with IBD microbiotas alone, HD mice alone, and in mice with IBD microbiotas that had received healthy DMT. IBD A, from a donor with Crohn’s disease, was notable for inducing a particularly high proportion of colonic Th17 cells in gnotobiotic mice (Fig. 2*B*) (15). In mice colonized with microbiota IBD A, DMT with all of the healthy microbiotas reduced the proportion of Th17 cells in the colon (Fig. 2 *B* and *C*). In mice colonized with IBD B or IBD C where baseline Th17 cells was lower, DMT did not significantly reduce the proportion of colon Th17 cells. A similar effect was observed in the ileum where the proportion of Th17 cells was reduced following four of the five DMTs in mice colonized with IBD A, but not significantly altered in mice colonized with IBD B or C (*SI Appendix*, Fig. S1).

## Identification of a Th17-Inducing Strain from a Donor with Crohn’s Disease

Intestinal Th17 cells can be induced by specific microbes, for example, SFB (17), a strain of *Bifidobacterium adolescentis* (27), and specific strains of *Escherichia coli* (18). As the proportion of Th17 cells induced by donor IBD A was particularly high, we hypothesized that the induction of Th17 cells by this microbiota was due to the presence of a specific strain that drives their induction and that perhaps the abundance of this strain was modulated following DMT, leading to the reduction in Th17 cells.

**Fig. 3.**
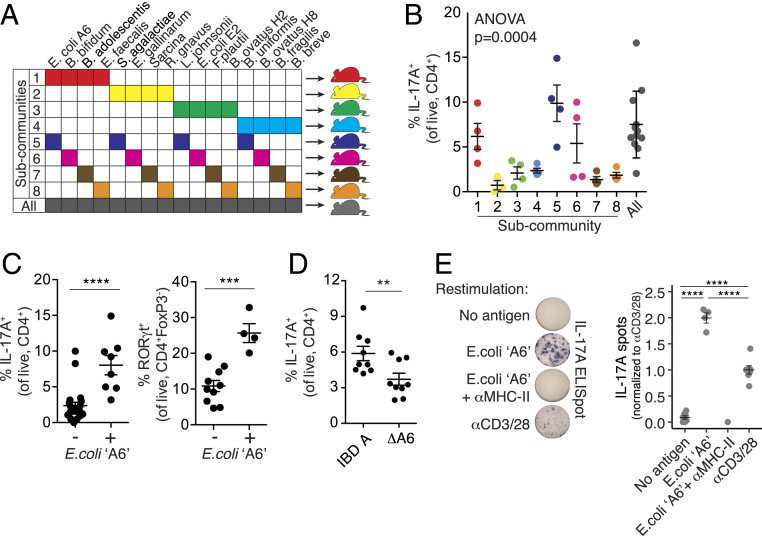
Identification of a Th17-inducing strain from a donor with Crohn’s disease. (*A*) Groups of germ-free B6 mice were each colonized with one of eight communities comprised of 4 of the 16 strains isolated from donor IBD A. (*B*) The proportion of IL-17A^+^ CD4^+^ T cells (of live CD4^+^ cells) in the colon lamina propria of mice colonized with each subcommunity derived from the IBD A microbiota. (*C*) The proportion of IL-17A^+^ CD4^+^ T cells and RORγt^+^ Th17 cells in mice colonized with communities in which *E. coli* strain A6 was present (+) or absent (−). (*D*) The proportion of IL-17A^+^ CD4^+^ T cells (of live CD4^+^ cells) in the colon lamina propria of mice colonized with the complete IBD A microbiota or a modified version of IBD A lacking *E. coli* strain A6 (ΔA6). (*E*) IL-17A secretion from mLN CD4^+^ T cells isolated from mice colonized with IBD A and restimulated ex vivo with anti-CD3 and anti-CD28 or dendritic cells loaded with *E. coli* strain A6, with or without an MHC-II blocking antibody. Plots show the mean and SE of each group of mice, and *P* values were calculated by *t* test (*C* and *D*), ANOVA (*B*), and ANOVA with Tukey correction (*E*). **P < 0.01, ***P < 0.001, ****P < 0.0001.

Using 16 strains from the IBD A microbiota, we assembled eight subcommunities, each with four strains, using an orthogonal design (28). Each strain was present in two different subcommunities. We colonized groups of germ-free B6 mice with the eight subcommunities and assessed the impact of each on the proportion of colon Th17 cells (Fig. 3*A*). The eight subcommunities induced varied proportions of IL-17A^+^ Th17 cells (*P* = 0.0004, ANOVA; Fig. 3*B*). We quantified the association of each strain with the proportion of Th17 cells and that found a single strain, *E. coli* A6, positively associated with the proportion of colon RORγt^+^ and IL-17A^+^ Th17 cells (*P* = 0.0035, *t* test; Fig. 3*C* and *SI Appendix*, Fig. S2*A*). Of note, a second strain of *E. coli*, isolated from IBD A (E2) was not associated with Th17 cells in this screen (*P* = 0.94, *t* test; *SI Appendix*, Fig. S2*A*). The effect appeared to be specific to RORγt^+^ effector T cells, as the proportion of RORγt^+^ FoxP3^+^ Treg cells was not associated with *E. coli* A6 (*SI Appendix*, Fig. S2*B*). To confirm the specific ability of *E. coli* A6 to induce Th17 cells, we generated a consortium of the strains isolated from IBD donor A excluding only *E. coli* A6. Mice colonized with this consortium lacking only *E. coli* A6 (ΔA6) had a significantly reduced proportion of colon IL-17A^+^ Th17 cells compared to mice colonized with the complete community from donor IBD A (Fig. 3*D*). These data suggested that *E. coli* A6 is required for Th17 cell induction in the context of microbiota IBD A. We next considered if *E. coli* A6 could have a similar immunomodulatory effect in a different setting. We constructed an artificial community of seven common commensal species (see *Methods* for details) and colonized groups of germ-free mice with this consortia with and without the addition of *E. coli* A6. In the context of this nonnative community, *E. coli* A6 significantly increased the proportion of lamina propria Th17 cells, but to a lesser extent than was observed for the original IBD-A community (*SI Appendix*, Fig. S2*C*). Strains of AIEC that induce Th17 cells in mice have been described (18). *E. coli* A6 was poorly invasive and adherent in a Caco-2 cell assay, did not survive in a macrophage cell line as compared to known strains of AIEC, and thus did not satisfy the phenotypic criteria for AIEC (*SI Appendix*, Fig. S2*D*). It was also negative for the pduC virulence factor previously associated with Th17 induction (18). These results demonstrate that non-AIEC *E. coli* strains can also induce Th17 cells.

CD4^+^ T cells from the mesenteric lymph nodes (mLN) of mice colonized with IBD A secreted IL-17A when restimulated ex vivo with dendritic cells loaded with a lysate of *E. coli* A6 (Fig. 3*E*) (29). This response was prevented by MHC-II blockade, suggesting a cognate interaction between the Th17 cells and DC-presenting antigen from *E. coli* A6 (Fig. 3*E*). Lamina propropria Th17 cells induced by the model Th17 inducing bacterium SFB are oligoclonal and characterized by an expansion of Th17 cells utilizing the Vβ14 T cell receptor (29). Using a broad panel of anti-Vβ chain antibodies, we tested if a similar expansion occurred in mice colonized with *E. coli* A6. In contrast to mice colonized with SFB, we did not find an expansion of Vβ14^+^ CD4 T cells in the Th17 compartment of the colon, small intestine, or mLN and did not see significant enrichment of any particular Vb chain in mice colonized with IBD A compared to SFB-containing microbiota (*SI Appendix*, Fig. S2*E*).

**Fig. 4.**
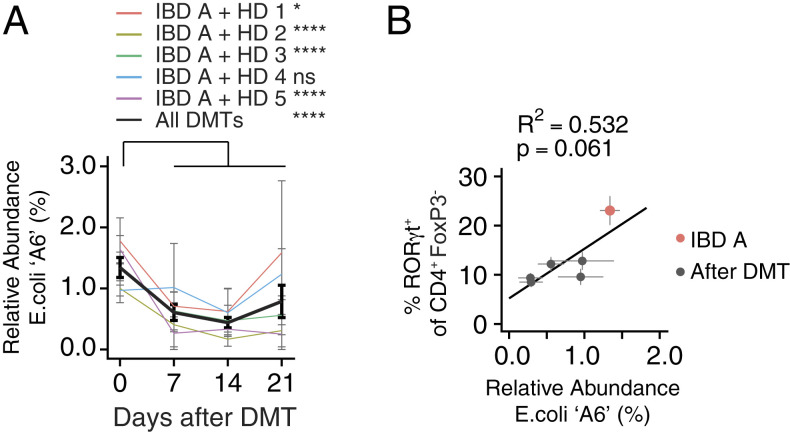
Modulation of an IBD-associated Th17-inducing strain following microbiota transplant. (*A*) The mean relative abundance of *E. coli* strain A6 from donor IBD A in mice before and at three time points after DMT with one of five HD microbiotas. (*B*) The correlation between the relative abundance of *E. coli* strain A6 and the proportion of RORγt^+^ Th17 cells in mice colonized with IBD A alone or following transplant with each of the five HD microbiotas. *P < 0.05, ****P < 0.0001 as calculated by ANOVA with Tukey correction comparing the relative abundance of the strain before transplant and at each time point after transplant. *P* value in *B* calculated by f-test.

We analyzed the relative abundance of the IBD-donor–derived strains in mice both before and following DMT with each of the five HD microbiotas. The abundance of the Th17-inducing *E. coli* A6 strain was significantly reduced in feces of mice following four of the five DMTs, relative to mice colonized with IBD A alone (Fig. 4*A* and *SI Appendix*, Fig. S3*A*). The relative and absolute abundance of *E. coli* A6 in the fecal microbiota before and after DMT was associated with the proportion of colon RORγt^+^ Th17 cells (Fig. 4*B* and *SI Appendix*, Fig. S3*B*). This suggests a mechanism whereby, in the context of the IBD A microbiota, DMT with HD microbiotas reduces the relative abundance of a specific strain, resulting in a reduction in lamina propria Th17 cells.

## Induction of Regulatory T Cells following DMT

**Fig. 5.**
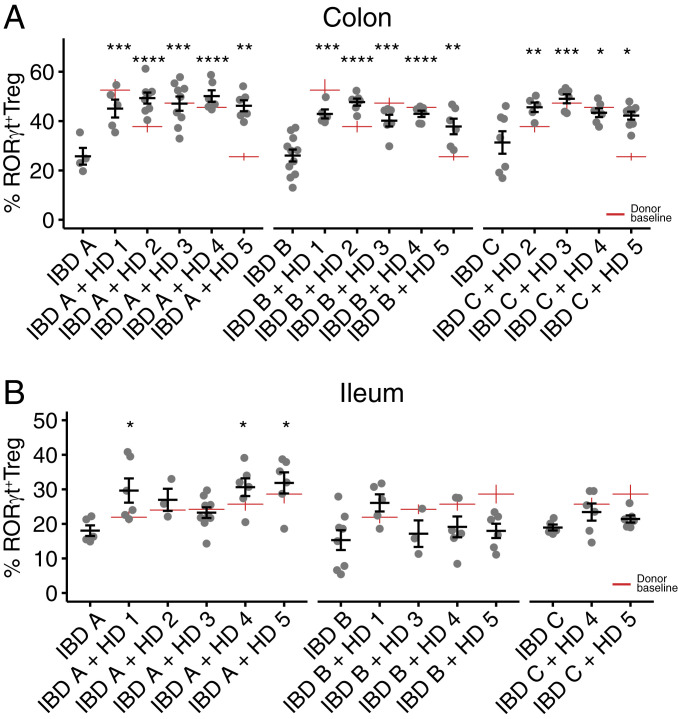
*ROR*γ*t*^*+*^ regulatory T cells are induced following defined microbiota transplant. (*A* and *B*) The proportion of colon and ileum lamina propria RORγt^+^ Treg cells (of live CD4^+^FoxP3^+^ cells) in groups of mice colonized with each IBD donor alone or 3 wk following DMT with one of the five HD microbiotas. Red lines indicate the proportion of RORγt^+^ Treg cells induced by each HD alone. Plots show the mean and SE of each group of mice. *P < 0.05, **P < 0.01, ***P < 0.001, and ****P < 0.0001 by ANOVA with Tukey correction.

The RORγt^+^ subset of intestinal regulatory T cells enforces tolerance to microbiota (30). RORγt^+^ Treg cells are dynamically and variably induced by different microbiotas (31) and are specifically deficient in mice colonized with microbiotas from donors with IBD (15). We hypothesize that one rational aim of microbiota-targeted therapy for IBD would be to increase the in situ differentiation and stability of RORγt^+^ Treg cells. When colonized alone, each of the three IBD recipient microbiotas (IBD A–C) induced a relatively low proportion of RORγt^+^ Treg cells in both colon and ileum of mice (Fig. 5 *A* and *B*). Three weeks following microbiota transplant, the proportion of RORγt^+^ Treg cells in the colon of recipient mice in all cases significantly increased relative to mice colonized with each IBD microbiota alone (Fig. 5*A*). We observed no “super donor” microbiota that consistently induced a higher proportion of RORγt^+^ Treg cells across all recipient microbiotas, and mice colonized with each of the three IBD donor microbiotas were similarly amenable to immune modulation by DMT in the colon.

Modulation of RORγt^+^ Treg cells was less pronounced in the ileum where significant increases were limited to three of the DMTs only in mice colonized with IBD A (Fig. 5*B*). In addition to the RORγt^+^ subset of Treg cells, we also observed modulation of the total gut FoxP3^+^ Treg population (*SI Appendix*, Fig. S4 *A* and *B*). Microbiotas that induce high proportions of RORγt^+^ Treg cells are also associated with reduced activation of colon dendritic cells (15, 30). Consistent with these observations, the expression of CD86 was lower on both total and CD11b^+^CD103^+^ (double positive) colonic dendritic cells from mice colonized with HD 1 than IBD A and are reduced post DMT (*SI Appendix*, Fig. S4*C*).

The proportion of total FoxP3^+^ Treg in colon and ileum was less consistently altered following DMT (*SI Appendix*, Fig. S4 *A* and *B*). Interestingly, the proportion of colon FoxP3^+^ Treg cells in each recipient+donor microbiota combination was predictable from the baseline induction by the donor microbiota alone (*R*^2^ = 0.47; *P* = 0.001, f-test) (*SI Appendix*, Fig. S4*D*). However, the proportion of RORγt^+^ Treg cells was not predictable from the baseline induction of these cells by the donor and recipient (*SI Appendix*, Fig. S4*E*). The observations of RORγt^+^ Treg cell induction following every tested DMT, and that the posttransplant proportion was unrelated to the proportion induced by the donors alone, suggested that RORγt^+^ Treg cell induction occurred by a mechanism unrelated to the specific composition of the microbiotas and may relate to broader structural characteristics of the communities.

## Microbiota Density Increases following Microbiota Transplant and Is Associated with Increased Mucosal Treg Cells in Mice and Endoscopic Remission in Individuals with UC

**Fig. 6.**
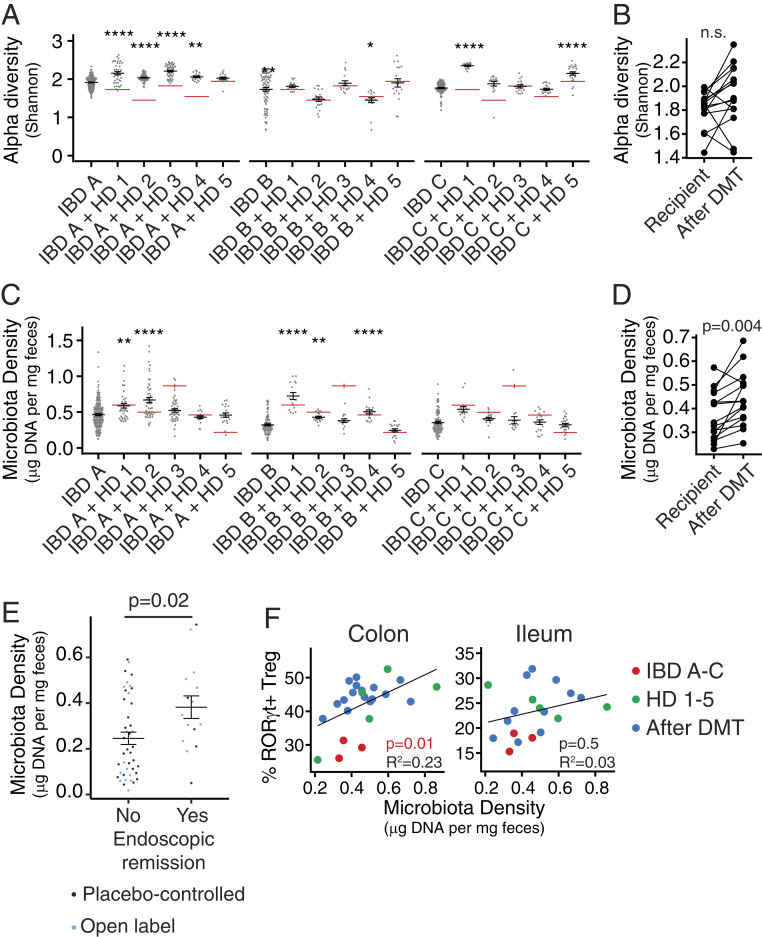
Microbiota density is increased following defined microbiota transplant and is associated with increased mucosal Treg cells in mice and endoscopic remission in individuals with UC following FMT. (*A*) The mean difference in fecal microbiota density in mice colonized with IBD microbiotas before and after DMT. Red lines indicate the fecal microbiota density in mice colonized with HD microbiotas alone. (*B*) Fecal microbiota density in groups of mice colonized with each IBD donor alone or 3 wk following DMT with one of the five HD microbiotas. (*C*) Alpha diversity of fecal microbiota in groups of mice colonized with each IBD donor alone or 3 wk following DMT with one of the five HD microbiotas. Red lines indicate the alpha diversity of fecal microbiota in mice colonized with HD microbiotas alone. (*D*) The mean difference in alpha diversity in mice colonized with IBD microbiotas before and after DMT. (*E*) Fecal microbiota density of FMT clinical trial participants with ulcerative colitis who did and did not achieve endoscopic remission following treatment in both the controlled placebo or open label arm of the trial. (*F*) The association between fecal microbiota density and the proportion of mucosal RORγt^+^ Treg cells in mice colonized with HD and IBD donor microbiotas alone or following DMT. In *A* and *C*, *P < 0.05, **P < 0.01, and ****P < 0.0001 by ANOVA with Tukey correction. Significance in *B* and *D* was calculated by paired *t* test, in *E* by unpaired *t* test, and in *F* by f-test.

The IBD microbiota has reduced alpha diversity (32, 33) and lower density than the microbiota from HDs (1, 23, 24, 34). The proportion of intestinal Treg cells can be influenced by microbiota composition (14–16, 21, 31), but can also be regulated independently of composition in response to changes in microbiota density (1). Following FMT for rCDI in humans, both the alpha diversity and the density of the gut microbiota increased (1, 35–38). We therefore examined if the increase in RORγt^+^Treg cells that we observe following DMT in our model could be attributed to changes in either alpha diversity or microbiota density (Fig. 6). We calculated microbiota density as the amount of DNA extracted from a fecal sample divided by the original sample mass (1, 39). Alpha diversity increased following some of the DMTs, particularly those involving IBD A and C, but this was not consistent for all tested pairs of IBD and HD microbiotas (*P* = 0.07, paired *t* test; Fig. 6 *A* and *B*). However, microbiota density significantly increased after DMT (*P* = 0.004, paired *t* test; Fig. 6 *C* and *D*.

Increased fecal microbiota density has been reported in individuals receiving FMT to treat rCDI (1). To assess the relevance of microbiota density modulation in FMT used in the context of IBD, we analyzed data from a recent placebo-controlled clinical trial of FMT in individuals with ulcerative colitis (9). This study reported endoscopic remission in ∼20% of individuals receiving an intensive multidonor FMT regime. Strikingly, microbiota density was higher following 8 wk of intensive FMT therapy in individuals in which endoscopic remission was achieved than in those where FMT did not lead to endoscopic remission (*P* = 0.024; Welch two sample *t* test, Fig. 6*E*).

In the gnotobiotic mouse model, microbiota density but not alpha diversity was significantly associated with the proportion of colonic RORγt^+^ Treg and total FoxP3^+^ Treg cells (*R*^2^ = 0.23, *P* = 0.01 and *R*^2^ = 0.14, *P* = 0.01, respectively, f-test; Fig. 6*F* and *SI Appendix*, Fig. S5 *A*–*C*). We find no association between microbiota density and the proportion of lamina propria Th17 cells (*SI Appendix*, Fig. S5*D*). A similar correlation between RORγt^+^ Treg and microbiota density was observed in mice colonized with a specific pathogen-free (SPF) microbiota and treated with different antibiotics. The greater the depletion of microbiota density following antibiotic treatment, the greater the reduction of mucosal RORγt^+^ Treg (*R*^2^ = 0.49, *P* < 0.0001, *R*^2^ = 0.11, *P* = 0.02 for colon and ileum, respectively; *SI Appendix*, Fig. S5*E*). Taken together, these data suggest that DMT with HD microbiotas can increase microbiota density in mice colonized with IBD donor microbiotas and in some humans receiving FMT for UC. In mice, the restoration of microbiota density correlates with the induction of gut Treg populations, and in humans with UC it is associated with endoscopic remission, suggesting reinstatement of tolerance in the intestinal tissues following transplantation.

## Mice Colonized with IBD Microbiotas Are Protected from Colitis by DMT

**Fig. 7.**
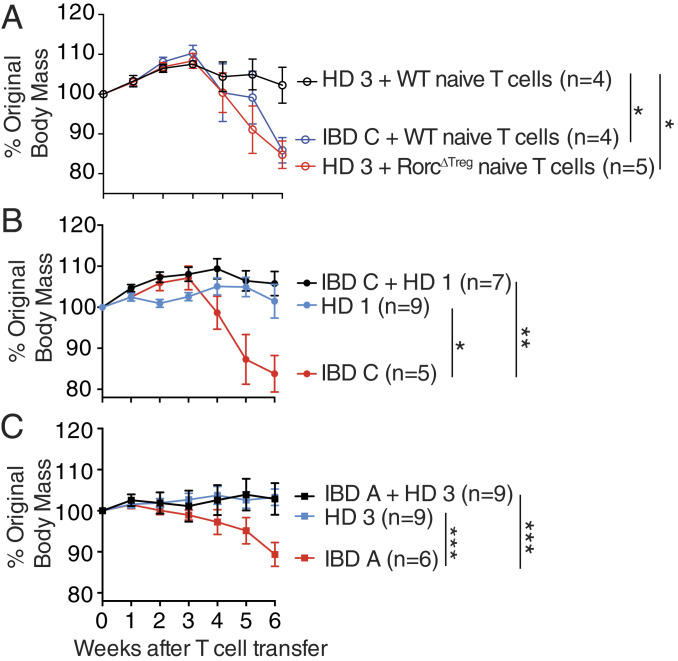
Mice colonized with IBD microbiotas are protected from colitis by DMT. (*A*) The change in body mass of Rag1^−/−^ mice colonized with the indicated microbiota following T cell transfer with cells of the indicated genotype. (*B* and *C*) The change in body mass following T cell transfer of Rag1^−/−^ mice colonized with (*A*) IBD C followed by DMT with HD 1 or (*B*) IBD A followed by DMT with HD 3. Shown are the mean and SEM of each group of mice, and the number of animals in each group is indicated in the plot. Data in each plot are combined from two independent experiments. *P < 0.05, **P < 0.01, and ***P < 0.001 by ANOVA with Tukey correction, comparing the change in mass of each group 6 wk after T cell transfer.

We have previously shown that induction of RORγt^+^ Treg cells is correlated with lower colitis severity in mice colonized with varied microbiotas (15) and that RORγt^+^ Treg cells protect from disease in mouse models of intestinal inflammation (31, 40, 41). We hypothesized that induction of RORγt^+^ Treg cells by the HD microbiotas is required to protect mice from colitis. To test this, we tried to induce colitis in mice colonized with a HD microbiota by transferring naive CD4^+^ T cells isolated from FoxP3-cre × Rorc^fl/fl^ (Rorc^ΔTreg^) mice, from which no RORγt^+^ Treg cells can develop, but in which RORγt^+^ effector Th17 cells are preserved (42). We found that transfer of Rorc^ΔTreg^ naive T cells led to more severe colitis than transfer of wild-type naive T cells, and in the absence of RORγt^+^ Treg cells, mice colonized with HD microbiota developed disease similar to mice colonized with an IBD microbiota (Fig. 7*A*). These data support the hypothesis that it is the capacity of a microbiota to induce RORγt^+^ Treg cells that is a major determinant of colitogenicity.

Based on this data, we hypothesized that the DMT interventions described above that induce RORγt^+^ Treg cells could protect mice from intestinal inflammation in a microbiota-dependent model of colitis. To test this, we colonized groups of Rag-deficient germ-free mice with microbiota IBD A or IBD C followed after 2 wk with a DMT of either microbiota HD-1 or HD-3. Colitis was induced after a further 2 wk by the transfer of naive (CD45RB^HI^ CD25^−^) CD4^+^ T cells, and colitis was monitored by changes in body mass. As expected (15), mice colonized with only an IBD microbiota developed more severe disease than those colonized with only a HD microbiota (Fig. 7 *B* and *C*). Mice that received DMT before colitis induction lost significantly less weight than those that did not receive DMT (Fig. 7 *B* and *C*), demonstrating that HD-derived DMT can protect from intestinal inflammation in this model.

## Discussion

Compositional changes to the gut microbiota are observed in many human diseases. Fecal microbiota transplantation is predicated on the assumption that an altered microbiota contributes to disease and restoring the composition of the microbiota to a “normal” state will provide therapeutic benefit. Despite a steady increase in FMT trials for different indications, we have little data to make predictions about how a treatment recipient will respond to a microbiota transplant and what are the desirable traits of a good donor and the molecular markers to indicate success beyond standard clinical end points. Manipulation of microbiota community characteristics, including colonization resistance, microbiota fitness, and microbiota density are likely key to the success of FMT in the context of recurrent *C. difficile* infection. However, the application of FMT in the context of autoimmune or inflammatory diseases likely requires achieving an element of specific immune modulation either in the gut, at a distal tissue site, or systemically. Immune modulation directed by microbiota manipulation could be achieved through a combination of suppression or elimination of proinflammatory effector strains and expansion or addition of tolerance-promoting strains or community characteristics. To advance microbiota manipulation as a potential therapeutic for immune-mediated disease, and to provide a solid scientific basis for the expanded use of such therapies beyond rCDI, we must better understand the potential of microbiota manipulation to alter specific immune populations and ultimately track these immune populations in FMT clinical trials (43–45).

We report a set of gnotobiotic mouse experiments that support the preclinical assessment of LBPs and microbiota transplants in the context of human inflammatory disease-associated microbiotas. In this study, we used mice colonized with defined IBD donor microbiotas and assessed how the immunophenotype of these hosts responds to DMT with HD microbiotas with known immunomodulatory properties across a total of 14 different microbiota transplants. The gut immune landscape of the mice is consistently reshaped following DMT. We demonstrate that introduction of HD microbiotas to mice colonized with IBD microbiotas can lead to an increase in the proportion of Treg cells, including the potently immunoregulatory RORγt^+^ Treg subset. We find that the increase in RORγt^+^ Treg cells occurs with a restoration of microbiota density following DMT. Microbiota density is decreased in humans with IBD and is correlated with the proportion of gut Treg cells in specific pathogen-free mice (1, 23). We found increased microbiota density in individuals with ulcerative colitis reaching endoscopic remission following intensive FMT in a recent placebo-controlled clinical trial (9), relative to individuals in whom endoscopic remission did not occur. We therefore hypothesize that the DMTs in mice restore the microbiota to a compositional or structural state that supports a greater microbiota density, which leads to induction of RORγt^+^ Treg cells. In humans receiving FMT for ulcerative colitis, a higher microbiota density following treatment associates with better endoscopic outcomes, suggesting a shift toward a more tolerance-promoting mucosal environment. Interestingly, the increase in RORγt^+^ Treg cells was not correlated with increases in alpha diversity, which is frequently associated with health and is one of the most commonly used metrics of dysbiosis. This suggests that microbiota density may be a metric of microbiota health as it relates to the tolerogenic Treg compartment and could be a useful biomarker for noninvasive monitoring of fecal microbiota transplant (1).

Microbiotas from donors with IBD induce a greater proportion of Th17 cells in the gut than do HD microbiotas (15, 18). Focusing on mice colonized with a microbiota from a donor with Crohn’s disease, we found that DMT using HD microbiotas suppressed the proportion of gut Th17 cells. This reduction in Th17 cells following DMT was not consistently associated with changes in microbiota density or alpha diversity. Instead, we found that a specific strain of *E. coli* was a primary driver of Th17 cell enrichment in mice colonized with this donor consortia and that decreased Th17 cells following DMT was correlated with a significant decrease in the abundance of this strain. Whereas RORγt^+^ Treg cell proportions were modulated by DMT following broad changes to the microbiota density, the reduction of Th17 cells following microbiota transplantation in mice colonized with this CD donor microbiota may be attributed to depletion of one specific strain. This more broadly reflects a different rationale being used to design microbiota-based therapeutics. Some approaches, such as FMT, aim to reverse dysbiosis and restore a broad structural and compositional state that is associated with health. Other approaches, such as the use of phage therapy or CRISPR/Cas-based strategies (4, 13), aim to target specific strains implicated in the particular disease. If microbiota-targeted therapies are to be successfully used in the context of complex diseases beyond rCDI, it is likely that a combination of these strategies will be required.

## Methods

### Human Microbiota Consortia.

Stool samples from IBD donor A (Crohn’s disease) and donors B and C (UC) were collected from de-identified donors in remission and were quickly frozen following donation before processing. Cultured consortia of microbes from human donor fecal samples IBD A–C and HD 1–4 were prepared as previously described (15). Briefly, a variety of solid media were inoculated with a slurry of each stool sample, and following incubation under anaerobic and aerobic conditions, liquid media (LYBHIv4 (46); 37 g/L Brain Heart Infusion [Becton Dickinson], 5 g/L yeast extract [Becton Dickinson], 1 g/L each of D-xylose, D-fructose, D-galactose, cellubiose, maltose, sucrose, 0.5 g/L *N*-acetylglucosamine, 0.5 g/L L-arabinose, 0.5 g/L L-cysteine, 1 g/L malic acid, 2 g/L sodium sulfate, 0.05% Tween 80, 20 μg/mL menadione, 5 mg/L hemin [as histidine-hemitin], 0.1 M Mops, pH 7.2) arrayed in multiwell plates were inoculated with the resulting colonies and cultures maintained under anaerobic conditions. Strains comprising each consortia were characterized by a combination of matrix-assisted laser desorption/ionization time-of-flight mass spectrometry, 16S ribosomal DNA amplicon and whole-genome shotgun sequencing. Strains were pooled in equal volumes and stored at −80 °C with 15% glycerol and defrosted immediately before inoculating mice. Microbiota HD 5 is a previously described consortia of predominantly clostridial species (14, 47). An artificial consortium of seven types of strains was used for some experiments. *B. ovatus* American Type Culture Collection (ATCC) 8483, *Bacteroides caccae* ATCC 43185, *Bacteroides thetaiotaomicron* ATCC VPI5482, *Bacteroides vulgatus* ATCC 8482, *Ruminococcus gnavus* ATCC 29149, *Clostridium bolteae* ATCC BAA-613, and *Collinsella aerofaciens* ATCC 25986 were grown in LYBHIv4 under anaerobic conditions and pooled in equal volumes before administering to mice (20). Isolated *E. coli* strains were grown in lysogeny broth **(**LB) in room air with shaking.

### Mice and Gnotobiotic Methods.

Germ-free C57BL/6J mice were bred in isolators at the Mount Sinai Precision Immunology Institute Gnotobiotic Facility. SPF C57BL/6J mice were purchased from Jackson Laboratories. Mice were colonized at approximately 6 wk old by a single gavage of a defined microbiota consortia. Following colonization, mice were housed in autoclaved filter-top cages, fed an autoclaved diet (5K67, LabDiet) and water, and handled under aseptic conditions. Some mice received a defined microbiota transplant, administered as a single gavage 3 wk after initial primary colonization. At the time of DMT, mice were transferred to a new autoclaved cage to minimize the carryover of the preexisting microbiota from the cage environment. Where indicated, mice were provided antibiotics at the following concentrations; ampicillin 1 mg/mL, ciprofloxacin 0.1 mg/mL, clindamycin 0.267 mg/mL, polymyxin B 0.1 mg/mL, or vancomycin 0.5 mg/mL in drinking water with 2% sucrose, prepared as previously described (1). FoxP3^YFP-cre^ (B6.129[Cg]-*Foxp3*^*tm4(YFP/icre)Ayr*^/J) and Rorc^fl/fl^ (B6[Cg]-*Rorc*^*tm3Litt*^/J) were purchased from Jackson Laboratories and crossed in-house under SPF conditions.

### Lymphocyte Isolation.

Gut tissues were prepared as previously described (15). Briefly, cleaned gut tissues were deepithelialized in 5 mM ethylenediaminetetraacetic acid (EDTA), 15 mM Hepes, and 5% fetal bovine serum (FBS) in Hank’s Balanced Salt Solution (HBSS) before digestion with 0.5 mg/mL Collagenase Type IV (Sigma Aldrich) and 0.25 mg/mL DNase 1 in HBSS with 2% FBS. Lymphocytes enriched by passing the cell suspension sequentially through 100- and 40-μm strainers. For enzyme-linked immune absorbent spot (EliSPOT) experiments, CD4^+^ T cells were isolated from mLN using magnetic isolation (CD4 microbeads, Miltentyi Biotech), and dendritic cells were isolated from the spleens of naive SPF C57BL/6 mice (Jackson Laboratories) by magnetic isolation (CD11c microbeads, Miltenyi Biotech) using an AutoMACS instrument (Miltenyi Biotech) following the manufacturer’s instructions.

### T Cell Transfer Colitis.

Colitis experiments were performed as previously described (15). Briefly, CD4^+^ T cells were magnetically enriched (Magnisort CD4 Enrichment Kits; Thermo Fisher) from spleens of naive SPF C57BL/6 (Jackson Labs), FoxP3^YFP-cre^ × Rorc^fl/fl^ mice, or FoxP3^YFP-cre^ × Rorc^WT/WT^ mice before fluorescence-activated cell sorting purification of CD4^+^ CD25^−^ CD45RB^HI^ cells. Rag1^−/−^ C57BL/6 mice were administered 1 × 10^6^ naive CD4 T cells by intraperitoneal injection and monitored for changes in body mass weekly.

### Flow Cytometry.

Flow cytometry of CD4^+^ T cells was performed as previously described (15). The following antibodies were used for T cell phenotyping: CD45 APC-Cy7 (Biolegend), CD4 APC (Biolegend), FoxP3 PE (Thermo Fisher/eBioscience), RORγt PerCp-Cy5.5 (BD Bioscience), and IL-17A-PE (Biolegend), and dead cells were excluded using Zombie Aqua (Biolegend). To detect intracellular IL-17A, isolated lymphocytes were first restimulated with 5 ng/mL phorbal 12-myristate 13-acetate and 500 ng/mL ionomycin in the presence of monensin (Biolegend) for 3.5 h. FoxP3 and RORγt expression was analyzed in unstimulated cells. For analysis of dendritic cells, the following antibodies were used: CD45 BrilliantViolet 750 (BD Bioscience), MHC-II Pacific Blue (Biolegend), CD11b PerCP-Cy5.5 (Biolegend), CD11c PE-Cy7 (Biolegend), CD64 BrilliantViolet 786 (BD Bioscience), and CD86 BrilliantViolet 605 (Biolegend), and dead cells were excluded with Fixable Viability Dye eFluor780 (ThermoFisher/eBioscience). The Vβ chain repertoire analysis used the following antibodies: Vβ2 AF647 (Biolegend), Vβ3 BrilliantViolet 510 (BD), Vβ5.1/5.2 PE-Cy7 (Biolegend), Vβ6 BrilliantViolet 650 (BD), Vβ7 FITC (Biolegend), Vβ8.1/8.2 APC-Vio770 (Miltenyi), Vβ10b BrilliantViolet 711 (BD), Vβ11 BrilliantViolet 421 (BD), Vβ12 BrilliantViolet 480 (BD), Vβ13 PerCp-eFluor710 (ThermoFisher/eBioscience), Vβ14 biotin (ThermoFisher/eBioscience) with streptavidin Qdot800 (ThermoFisher), Vβ17a BrilliantViolet 605 (BD), CD45 BrilliantViolet 750 (BD), FoxP3 PE (ThermoFisher/eBioscience) RORγt APC (BD) and CD4 BrilliantViolet 570 (Biolegend). T cell phenotyping data were generated using an LSRII instrument (BD Biosciences). Vβ chain and dendritic cell phenotype data were acquired using an Aurora spectral cytometer (Cytek). Baseline immune profiles for the three recipient communities and four of the five donor communities (except donor 5) were previously published (15) and are included here as baseline reference points to compare with the results of the DMT.

### EliSPOT Assay.

EliSPOT experiments for the detection of microbe-specific T cell responses were performed as previously described with some minor modifications (29). CD4^+^ T cells were isolated from the mLN of gnotobiotic mice using magnetic isolation (CD4 microbeads, Miltentyi Biotech), and dendritic cells were isolated from the spleens of naive SPF C57B/6 mice (Jackson Laboratories) by magnetic isolation (CD11c microbeads, Miltenyi Biotech) using an AutoMACS instrument (Miltenyi Biotech) following the manufacturer’s instructions. Bacterial strains were grown to stationary phase in LYBHIv4 media, and bacterial antigen was prepared by autoclaving the washed bacteria in phosphate-buffered saline (PBS). Dendritic cells were plated at a density of 50,000 cells/well and pulsed with antigen (1:100 dilution of the stationary phase culture) overnight. Isolated CD4^+^ T cells were added to the culture the next day (2:1 T cell to dendritic cell ratio) and placed at 37° for 48 h. Where indicated, MHC class II blocking antibody (M5/114.15.2, Biolegend, 2.5 μg/mL) was added to DC cultures 1 h prior to adding the CD4^+^ T cells. After 48 h of coculture, the cells were transferred to an IL-17A ELISPOT plate for an additional 24 h and then developed according to the manufacturer’s instructions (R&D Systems).

### Genome and Metagenome Sequencing.

DNA was extracted from isolated bacterial strains by mechanical dissociation by bead beating in 282 μL of DNA buffer A (20 mM Tris, pH 8.0, 2 mM EDTA, and 200 mM NaCl), 200 μL of 20% sodium dodecyl sulfate (SDS) (v/w), 550 μL of Phenol:Chloroform:IAA (25:24:1), 268 μL of Buffer PM (Qiagen), and 400 μL of 0.1-mm-diameter zirconia/silica beads (20). DNA was extracted from mouse feces by bead beating in DNase Inactivation Buffer (0.5% SDS, 0.5 mM EDTA, 20 mM Tris, pH 8.0, with 200 μL of 0.1-mm-diameter zirconia/silica beads (1). DNA from bacterial isolates and feces was isolated using QiaQuick (Qiagen) columns and quantified by Qubit assay (Life Technologies). Sequencing libraries were generated from sonicated DNA with the NEBNext Ultra II DNA Library Prep kit (New England BioLabs). Ligation products were purified with SPRIselect beads (Beckman Coulter), and enrichment PCR was performed with NEBNext Ultra Q5 Master Mix (New England BioLabs). Samples were pooled in equal proportions and size-selected using 0.6× followed by 0.2× of AMPure XP beads (Beckman Coulter) before sequencing with an Illumina HiSeq (paired-end 150 bp). Reads from metagenomic samples were trimmed, subsampled to 100,000 reads, and mapped to the unique regions of bacterial genomes known to potentially form part of the microbiota in a given gnotobiotic sample. Genomes for strains in HD-5 were downloaded from NCBI; accession numbers can be found in Dataset S1 (14). Abundances were scaled to the size of each specific genome as previously described (48). Microbiota density was calculated as the mass of DNA extracted from a fecal sample, divided by the mass of the sample (1).

### *E. coli* Adhesion, Invasion, and Motility Assays.

Colonic epithelial cell line Caco-2 (ATCC HTB-37) and murine macrophage cell line J774 (ATCC TIB67) were obtained from ATCC and grown according to ATCC protocols. Caco-2 cells were grown in 24-well plates for 2 d, and confluent monolayers were infected with *E. coli* strains at a multiplicity of infection (MOI) of 10 for 3 h. For adhesion assays, cells were washed three times with PBS at 3 h postinfection, serially diluted, and plated on LB agar. For invasion, cells were washed three times with PBS, and extracellular bacteria were killed with 100 µg/mL gentamicin for 1 h. Intracellular bacteria were enumerated as described above. The adhesion and invasion levels were expressed as the total number of colony-forming units per milliliter recovered per well. J774 macrophages were seeded in 24-well plates at a density of 2 × 10^5^ cells/mL. The next day they were infected with *E. coli* at a MOI of 10 for 1 h. After the infection period, cells were washed three times with PBS, and extracellular bacteria were killed with 100 µg/mL gentamicin before gentamicin concentration was dropped to 20 µg/mL for longer infection periods. Survival after 24 h was expressed as the mean percentage of bacteria recovered after 1 h post infection, defined as 100%. For cell culture assays, a noninvasive *E. coli* strain (DH5α) and an AIEC strain CU 541–15 or LF82 were used as negative and positive controls, respectively (49). To determine the motility of the *E. coli* strains, *E. coli* was grown overnight at 37 °C in LB broth. Two microliters of overnight culture was inoculated onto soft agar (1% tryptone, 0.5% NaCl, 0.25% agar), and plates were incubated at 30 °C overnight. Motility was determined by measuring the diameter of the ring formed by each strain.

## Supplementary Material

Supplementary File

Supplementary File

## Data Availability

Anonymized next-generation sequencing (NGS) data have been deposited in the National Center for Biotechnology Information under accession nos. PRJNA518912 and PRJNA589044.
