# Supplementary material for: Defined microbiota transplant restores Th17/RORγt+ regulatory T cell balance in mice colonized with inflammatory bowel disease microbiotas
Source: Proc Natl Acad Sci U S A. 2020 Aug 18;117(35):21536–45. doi: 10.1073/pnas.1922189117 (PMC7474624; doi:10.1073/pnas.1922189117)
Supplement: Supplementary File [file pnas.1922189117.sapp.pdf]

PNAS
www.pnas.org

Supplementary Information for

Defined microbiota transplant restores Th17/RORγt^{+} regulatory T cell balance in mice colonized with inflammatory bowel disease microbiotas

Graham J. Britton, Eduardo J. Contijoch, Matthew P. Spindler, Varun Aggarwala, Belgin Dogan, Gerold Bongers, Lani San Mateo, Andrew Baltus, Anuk Das, Dirk Gevers, Thomas J. Borody, Nadeem O. Kaakoush, Michael A. Kamm, Hazel Mitchell, Sudarshan Paramsothy, Jose C. Clemente, Jean-Frederic Colombel, Kenneth W. Simpson, Marla C. Dubinsky, Ari Grinspan and Jeremiah J. Faith

Jeremiah J. Faith
Email: jeremiah.faith@mssm.edu or
Graham J. Britton
Email: graham.britton@mssm.edu

This PDF file includes:

Figures S1 to S5
Legends for Datasets S1

Other supplementary materials for this manuscript include the following:

Datasets S1

www.pnas.org/cgi/doi/10.1073/pnas.1922189117

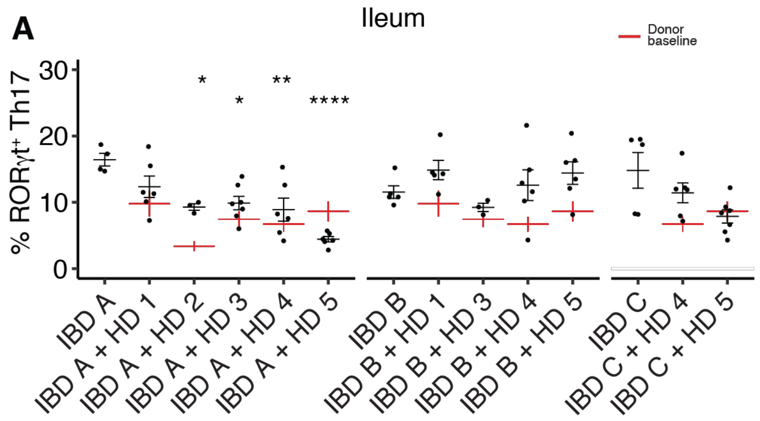

Fig S1. Modulation of mucosal Th17 cells by defined microbiota transplant. (A) The proportion of RORγt+ Th17 cells (of live, $CD4^+FoxP3^-$ cells) in the ileum lamina propria of gnotobiotic mice colonized with the five HD-derived microbiotas used as DMT donors. Red lines indicate the proportion of Th17 cells induced by each HD alone. Plots show the mean and standard error of each indicated group of mice. *$p<0.05$, **$p<0.01$, ****$p<0.0001$ by ANOVA with Tukey correction.

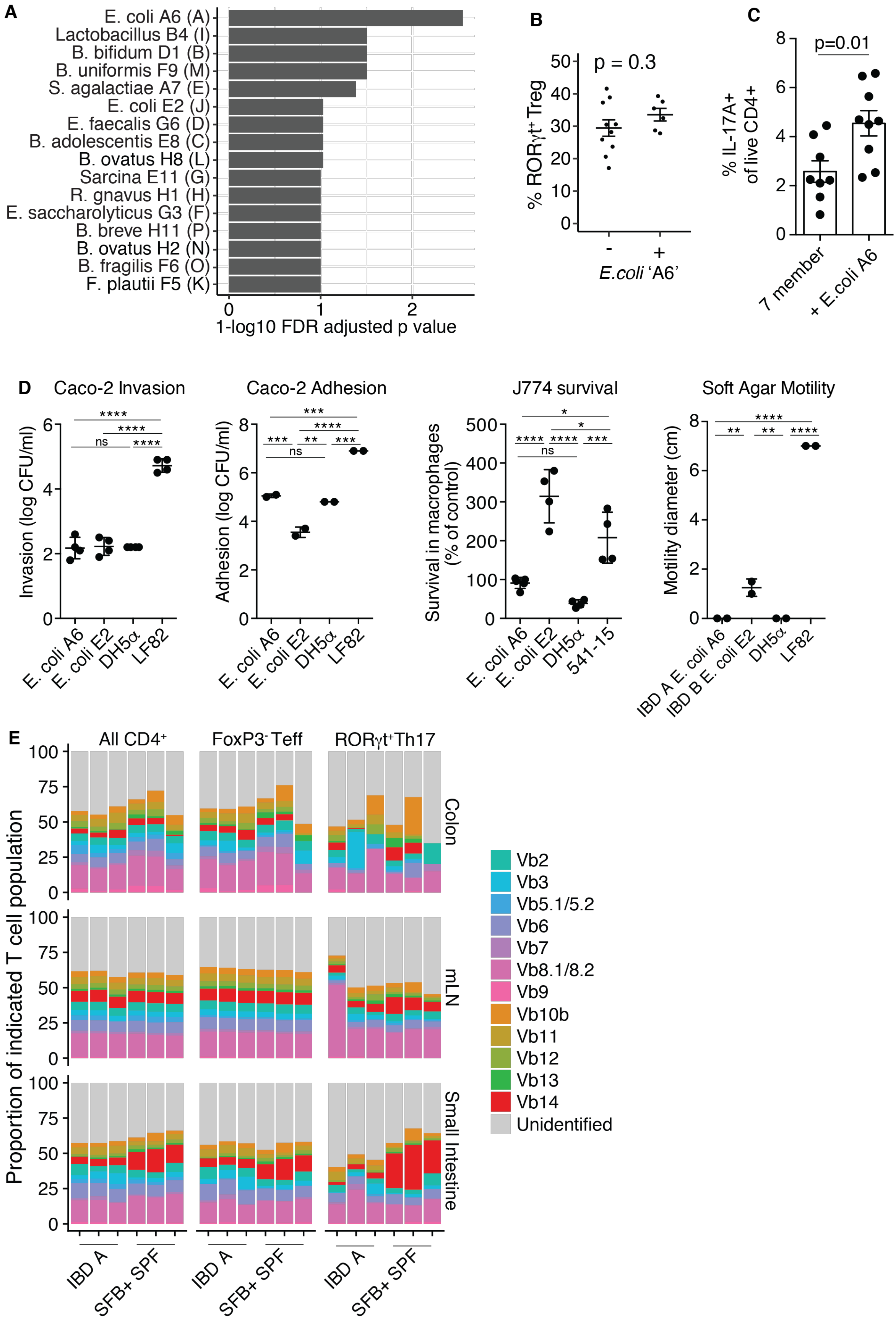
A
E. coli A6 (A)
Lactobacillus B4 (I)
B. bifidum D1 (B)
B. uniformis F9 (M)
S. agalactiae A7 (E)
E. coli E2 (J)
E. faecalis G6 (D)
B. adolescentis E8 (C)
B. ovatus H8 (L)
Sarcina E11 (G)
R. gnavus H1 (H)
E. saccharolyticus G3 (F)
B. breve H11 (P)
B. ovatus H2 (N)
B. fragilis F6 (O)
F. plautii F5 (K)
1-log10 FDR adjusted p value
B
p = 0.3
% RORγt+ Treg
E.coli 'A6'
C
p=0.01
% IL-17A+ of live CD4+
7 member
+ E.coli A6
D
Caco-2 Invasion
Invasion (log CFU/ml)
Caco-2 Adhesion
Adhesion (log CFU/ml)
J774 survival
Survival in macrophages (% of control)
Soft Agar Motility
Motility diameter (cm)
E. coli A6
E. coli E2
DH5α
LF82
541-15
IBD A E. coli A6
IBD B E. coli E2
E
All CD4+
FoxP3- Teff
RORγt+Th17
Proportion of indicated T cell population
Colon
mLN
Small Intestine
IBD A
SFB+ SPF
Vb2
Vb3
Vb5.1/5.2
Vb6
Vb7
Vb8.1/8.2
Vb9
Vb10b
Vb11
Vb12
Vb13
Vb14
Unidentified

Fig S2. Identification of a Th17-inducing strain from a donor with Crohn's disease. (A) FDR-adjusted one-tailed p values for the positive association of each strain from defined microbiota IBD A with the proportion of colon IL-17A^{+} CD4 T cells derived from the *in vivo* gnotobiotic screen described in Figure 3. Data is combined from 2 independent experiments. (**B**) The proportion of RORγt^{+} Treg cells in colon lamina proporia of mice colonized with communities in which E. coli strain A6 was present (+) or absent (-). (**C**) The proportion of IL-17A^{+} CD4 T cells induced in the colon lamina propria of mice colonized with a synthetic community of seven commensals (see methods for details) with or without the addition of *E. coli* A6. Each point represents data from one mouse, bars indicate the mean +/- SEM of each group. Data in A and B combined from 2 independent experiments. (**D**) The ability of *E. coli* A6 and E2 to adhere to and invade Caco-2 cells, persist in J774 macrophage cells and migrate on soft agar relative to a negative control strain (DH5a) or positive control strain (LF82 or 541-15). (**E**) Vβ chain usage by CD4^{+} T cells from mLN and colon and small intestine lamina propria of mice colonized with IBD A or an SFB-containing SPF microbiota. Each bar shows data from one mouse. P value in (B) and (C) calculated by unpaired t-test and in (D) by ANOVA with Tukey correction.

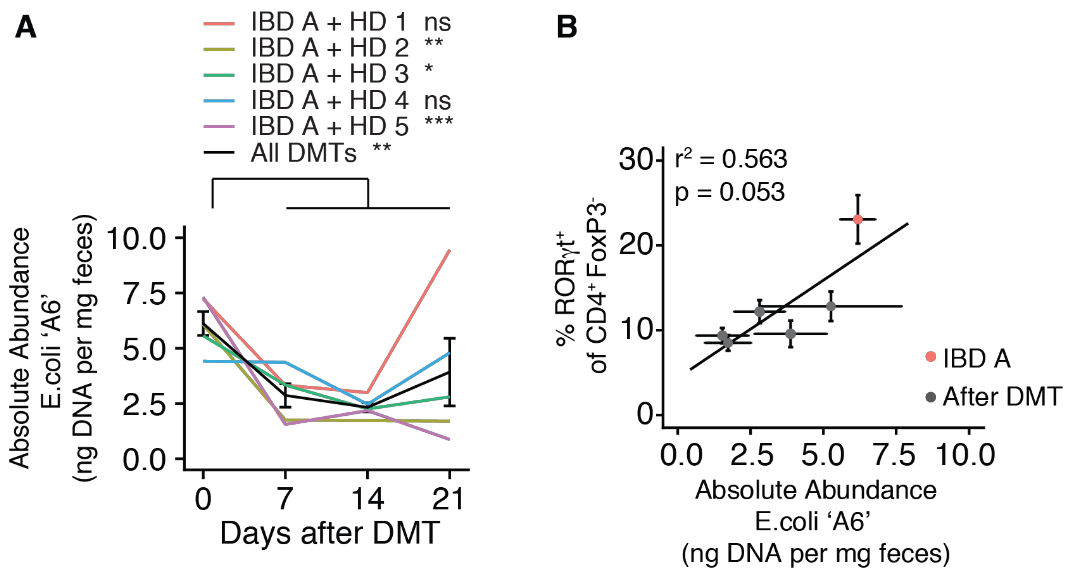

Fig S3. Modulation of the absolute abundance of an IBD-associated Th17-inducing strain following microbiota transplant. (A) The mean absolute abundance of *E. coli* strain A6 from donor IBD A in mice before and at three timepoints after DMT with one of five HD microbiotas. (**B**) The correlation between the absolute abundance of E. coli strain A6 and the proportion of RORγt+ Th17 cells in mice colonized with IBD A alone, or following transplant with each of the five HD microbiotas. *p<0.05, **p<0.01, ***p<0.001 as calculated by ANOVA with Tukey correction comparing the relative abundance of the strain before transplant and at each timepoint after transplant in (A) and by f-test in (B).

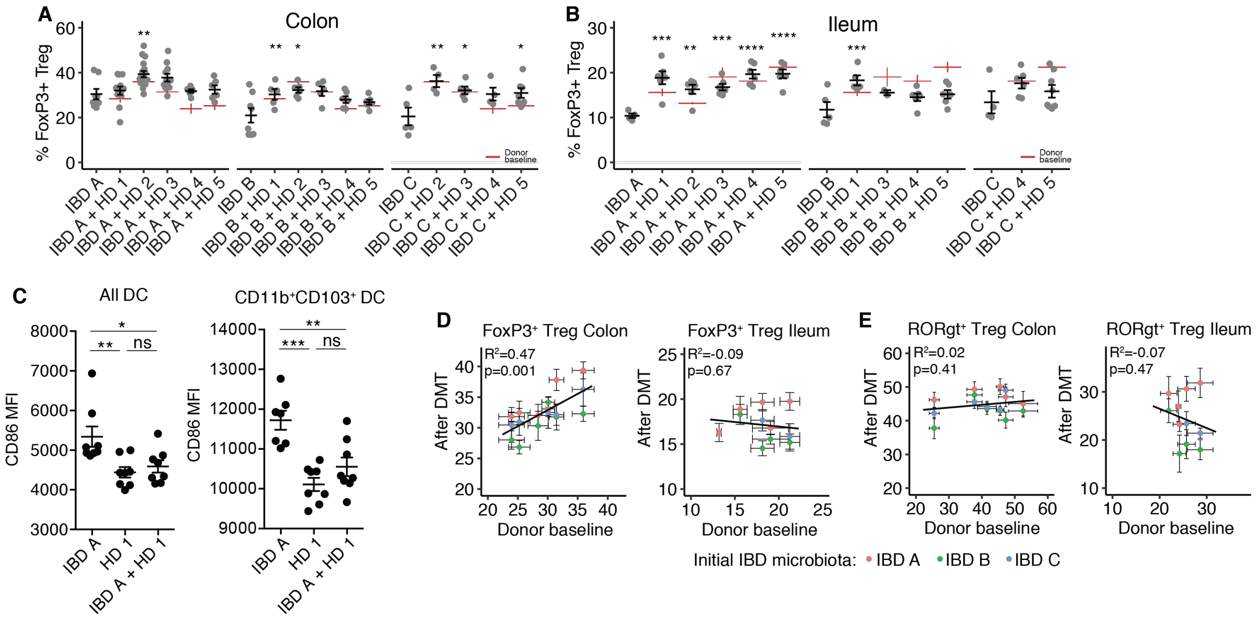

Fig S4. Modulation of FoxP3+ Treg cells and mucosal dendritic cell phenotype following microbiota transplant. (A-B) The proportion of colon and ileum lamina propria total FoxP3+ Treg cells (of live, CD4+ cells) in groups of mice colonized with each IBD donor alone or three weeks following DMT with one of the five HD microbiotas. Red lines indicate the proportion of FoxP3+ Treg cells induced by each HD alone. (**C**) The expression of CD86 on total dendritic cells (MHC-II+, CD64-, CD11c+) and double-positive dendritic cells (MHC-II+, CD64-, CD11c+, CD11b+, CD103+) in colon of mice colonized with donor IBD A or HD 1 alone, or following microbiota transplant with HD 1. (**D-E**) The association between the proportion of the indicated cell types in mice colonized with a HD microbiota alone, or in mice receiving the HD microbiota as a transplant following with one of three IBD microbiotas. *p<0.05, **p<0.01, ***p<0.001, ****p<0.0001, by ANOVA with Tukey correction (A-C) or f-test (E and E), ns – not significant.

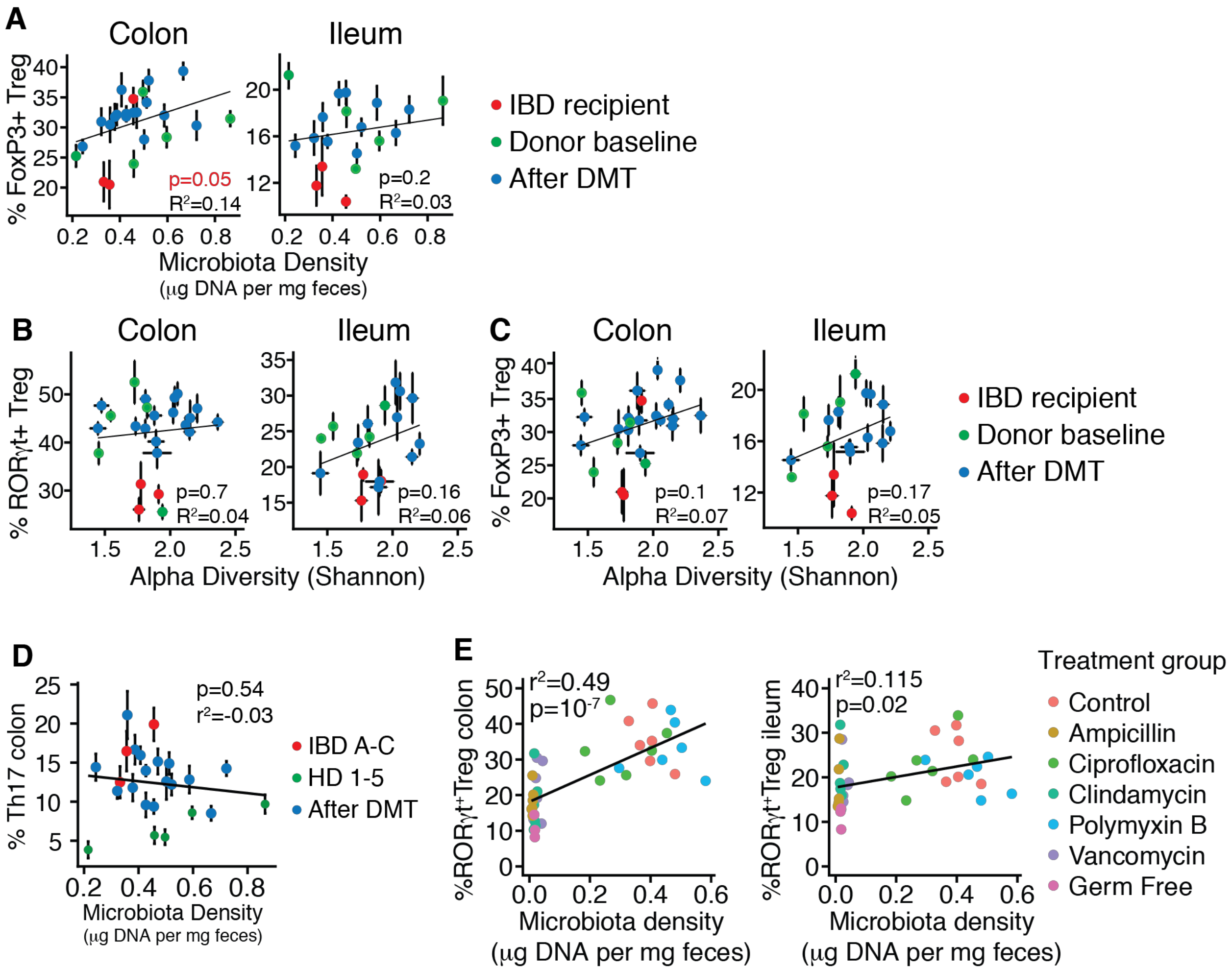

Fig S5. The association of microbiota diversity and density with mucosal RORγt+ Treg cells. (**A**) The association between fecal microbiota density and the proportion of mucosal FoxP3+ Treg cells in mice colonized with HD and IBD donor microbiotas alone or following DMT. (**B-C**) The association between fecal microbiota alpha diversity and the proportion of mucosal RORγt+ Treg and FoxP3+ Treg cells in mice colonized with HD and IBD donor microbiotas alone or following DMT. (**D**) The association between fecal microbiota density and the proportion of mucosal RORγt+ Th17 cells in mice colonized with HD and IBD donor microbiotas alone or following DMT. (**E**) The association between fecal microbiota density and the proportion of mucosal RORγt+ Treg cells in specific pathogen free mice treated with the indicated antibiotic in drinking water. P values calculated by by f-test.

Dataset S1 (separate file). A list of the strains comprising each consortium.

1.
